# Ternary-code DNA methylation dynamics: A new era for scalable mapping

**DOI:** 10.1016/j.xgen.2025.101006

**Published:** 2025-09-10

**Authors:** Yubin Zhou, Yun Huang

**Affiliations:** 1 Center for Translational Cancer Research, Institute of Biosciences and Technology, Texas A&M University, Houston, TX, USA; 2 Department of Translational Medical Sciences, College of Medicine, Texas A&M University, Houston, TX, USA; 3 Center for Epigenetics and Disease Prevention, Institute of Biosciences and Technology, Texas A&M University, Houston, TX, USA; 4 Department of Biomedical Engineering, College of Engineering, Texas A&M University, College Station, TX, USA

## Abstract

The preview discusses the scalable platform for methylation-based trait mapping published in *Cell Genomics* by Goldberg et al.^1^ This work represents not only a methodological advance but also marks a conceptual shift toward scalable and high-throughput functional, targeted, and context-sensitive epigenomic screening.

## Teaser

The preview discusses the scalable platform for methylation-based trait mapping published in *Cell Genomics* by Goldberg et al. This work represents not only a methodological advance but also marks a conceptual shift toward scalable and high-throughput functional, targeted, and context-sensitive epigenomic screening.

## Main text

Epigenome-wide association studies (EWASs) play a critical role in uncovering how dynamic epigenetic marks intersect with complex human traits and diseases.^2^ Among the many epigenetic marks, DNA methylation, particularly 5-methylcytosine (5mC), has been the primary focus owing to its dynamic, context-dependent regulation of transcription, its role in mediating gene-environment interactions, and its utility as a biomarker of developmental history, lifestyle, and disease.^3^ Microarray-based profiling has powered much of this progress, with Illumina’s Infinium BeadChip platform supporting thousands of EWAS publications and enabling widely used predictors such as the Horvath epigenetic clock, disease risk indices, and environmental exposure scores.^4^^,^^5^ However, EWAS approaches have been limited by technical constraints. Firstly, probe selection has favored broad genomic coverage rather than optimal capture of trait-associated or cell-type-specific cytosine-phosphate-guanine dinucleotide motifs (CpGs), leaving critical regulatory regions underrepresented. Secondly, standard bisulfite-based workflows cannot distinguish between 5mC and its oxidized derivative, 5-hydroxymethylcytosine (5hmC), an epigenetic mark essential for DNA demethylation, enriched at active regulatory elements and increasingly recognized as a stable and functionally distinct signal with its own regulatory roles in development, aging, and disease.^6^^,^^7^ While sequencing-based methods, including whole-genome bisulfite sequencing (WGBS), reduced representation bisulfite sequencing (RRBS), and single-cell methylome profiling (joint-snhmC-seq^8^ and SIMPLE-seq^9^) can resolve these modifications, their high cost, relatively limited throughput, and analytical complexity have hindered adoption in large-scale population studies. Even the updated Illumina Infinium Human MethylationEPIC version 2.0 BeadChip array (EPIC v2), with expanded enhancer coverage and compatibility with epigenetic clocks, did not address the need for scalable and joint profiling of 5mC and 5hmC.

**Figure 1:**
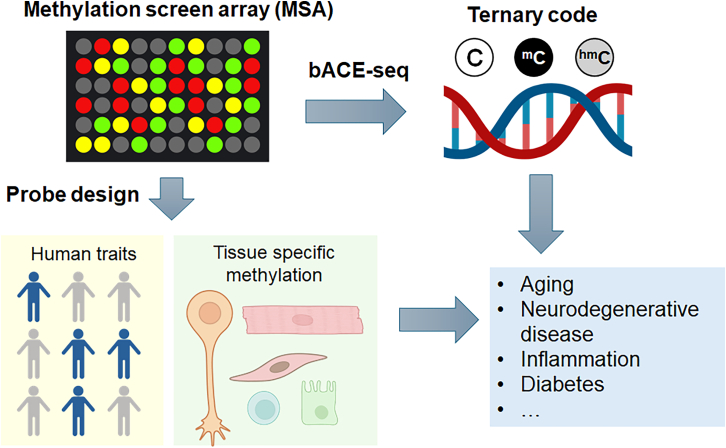
The methylation screening array includes customized probes that cover human traits and tissue-specific methylation information When combined with bACE-seq, which distinguishes between methylated and hydroxymethylated cytosines (5mC and 5hmC), this platform enables the discovery of DNA-methylation changes associated with human traits in a scalable manner.

In this issue of *Cell Genomics*, Goldberg et al. introduce the methylation screening array (MSA), a next-generation Infinium BeadChip that directly addresses these long-standing limitations (Figure 1). The MSA is specifically tailored for trait-centric EWAS and cell-type deconvolution, with the additional ability to profile both 5hmC and 5mC via a bisulfite-apolipoprotein B mRNA editing catalytic polypeptide-like protein family (APOBEC) workflow (“bACE”).^1^^,^^10^ This combination of targeted, biologically enriched content and ternary methylation code (5mC, 5hmC, unmodified C) detection marks a conceptual shift in array-based epigenomics, which promises to diminish the gap between high-resolution mechanistic maps and cost-effective population studies. The MSA represents a thoughtful departure from previous methylation-array designs. Rather than offering wide but even genomic coverage, the authors deliberately condense probe content to emphasize trait-associated and cell-type-specific CpG sites. This strategy turns out to be both practical and powerful. The authors curated and prioritized probes from over 1,000 EWAS publications, public databases, and high-resolution single-cell and bulk methylome studies. These efforts led to a compact array with 284,317 probes, which is significantly fewer than EPICv2 but with greater enrichment for trait-associated sites by expanding inclusion of cell-identity-defining methylation signatures. Compared to EPICv2, MSA exhibits improved representation of enhancer elements and cell-type-discriminating loci, including rare and specialized cell types such as neuronal subpopulations. The array also enables high-throughput profiling via a 48-sample format, thus making it well suited for large-scale population studies. Importantly, while the design omits a number of legacy EPIC probes, the authors provide robust strategies for probe imputation and cross-platform compatibility to ensure that predictive models such as epigenetic clocks remain interpretable and useful.

A major innovation of this study lies in the scalable dissection of DNA methylation into 5mC and 5hmC components, traditionally indistinguishable in standard bisulfite-based arrays. By combining MSA with the bACE-seq protocol (bisulfite + APOBEC3A deamination),^10^ the authors generate matched 5modC (cytosine modifications, 5mC + 5hmC) and 5hmC profiles from the same tissue samples. The resulting data offer a more complete, ternary epigenetic landscape across 117 human tissue samples and 64 whole blood profiles, which are valuable resources in the field. Notably, the study reveals that 5hmC patterns are highly tissue specific; are enriched in brain, kidney, and liver; and are depleted in mitotically active tissues such as colon and skin. Consistent with previous publications, 5hmC marks are largely positively correlated with gene expression, localize to promoters and enhancers, and complement the silencing roles of 5mC.^6^ This division of labor suggests that 5hmC serves not only as a demethylation intermediate but as a stable and functional mark, particularly in post-mitotic and developmentally specialized cell types. The authors further demonstrate that 5hmC accumulation is positively associated with chronological age at specific loci, often overlapping with canonical epigenetic clock sites. Remarkably, 5hmC alone can predict age with accuracy comparable to 5mC-based models. This finding raises intriguing questions about the biological meaning of epigenetic aging signals: are they capturing gene repression, stable activation, or both?

A major strength of MSA is its capacity to situate EWAS and GWAS findings within their tissue and cell-type context. By profiling diverse tissues and applying sophisticated computational deconvolution tools, the authors demonstrate that trait-associated CpG sites are often most variably methylated in the relevant tissues. For instance, CpGs associated with Alzheimer’s disease are most altered in brain tissue, while irritable-bowel-syndrome-related ones are most dynamic in the colon and small intestine. Similarly, GWAS variants linked to glucose levels and diabetes colocalize with methylation markers of pancreatic cell types, reinforcing the importance of tissue-aware interpretation of methylation data. This context-resolving capacity is further enhanced by its expanded cell-type marker catalog, which includes over 170 distinct cell types derived from single-cell and sorted-cell WGBS data. These features enable accurate deconvolution of bulk samples, revealing predictable shifts in immune-cell composition with age and sex as well as individual-level variability across 64 whole blood donors. Congruently, these data point toward a future where EWAS interpretation is deeply rooted in cellular architecture and differentiation state.

Beyond trait mapping, the study highlights previously underappreciated biological signals in the human methylome. The authors identify a set of intermediately methylated CpGs across tissues that likely correspond to imprinted loci, some known (e.g., PEG10 and GNAS), others potentially novel. They also reveal over 1,800 sex-associated CpG sites, including nearly 1,000 on autosomes, with pronounced 5mC and 5hmC differences between males and females. These findings may have implications for understanding sex-biased gene expression and disease susceptibility. Of particular interest is the demonstration that epigenetic clocks, traditionally trained on total 5modC profiles, may inadvertently be capturing 5hmC-driven aging dynamics. By comparing 5mC-only, 5hmC-only, and combined 5modC clocks, the authors show that 5hmC contributes measurably to age prediction, suggesting that future clock models could benefit from explicitly modeling the ternary methylation code.

While this work is largely focused on validation and design benchmarking, future studies using MSA could provide new insights into population epigenetics, disease-risk stratification, and cell-type-specific regulatory mechanisms. The affordability and throughput offered by MSA also make it a strong candidate for use in longitudinal cohorts, multi-omic consortia, and global health studies, especially those involving ancestrally diverse populations historically underrepresented in EWASs. By enabling routine, large-scale measurement of 5hmC alongside 5mC, the MSA platform brings hydroxymethylation into the mainstream of EWASs, thereby opening the door to novel biological insights in development, aging, and diseases. Its enrichment for tissue-relevant CpGs enhances the mapping between traits and the tissues in which their epigenetic signatures are most pronounced, thus increasing the mechanistic interpretability of EWAS findings. The integration of trait-linked CpGs with genome-wide association study signals promises to not only strengthen causal inference but also improve the functional annotation of disease-associated loci. Importantly, the cost parity with existing methylation arrays makes the platform feasible to apply in larger cohorts at population scale.

In summary, Goldberg et al. have delivered a scalable platform that addresses many of the persistent challenges in methylation-based trait mapping: cell-type heterogeneity, incomplete modification detection, and limited representation of diverse traits and populations. The MSA represents not only a methodological advance but also a conceptual shift toward functional, targeted, and context-sensitive epigenomic screening in a scalable and high-throughput manner.

## Declaration of interests

The authors declare no competing interests.
